# Systematic Review of Chinese Traditional Exercise Baduanjin Modulating the Blood Lipid Metabolism

**DOI:** 10.1155/2012/282131

**Published:** 2012-11-12

**Authors:** Lijuan Mei, Qingyue Chen, Li Ge, Guohua Zheng, Jinxiu Chen

**Affiliations:** ^1^School of Nursing, Fujian University of Traditional Chinese Medicine, Fuzhou 350122, China; ^2^Institute of Integrative Medicine, Fujian University of Traditional Chinese Medicine, Fuzhou 350122, China

## Abstract

*Background*. Baduanjin exercise is considered to be beneficial to modulate the blood lipid metabolism. The purpose of the systematic review was to assess the potential efficacy and safety of Baduanjin exercise. *Methods*. MEDLINE, EMBASE, CBM, CNKI, VIP, Chinese Important Conference Papers Database, and Chinese Dissertation Database were searched for all prospective-controlled trials of Baduanjin exercise from their inception to December 31, 2011. *Results*. A total of 14 studies were included. Comparing with no treatment, Baduanjin exercise significantly reduced the levels of TC, TG, LDL-C in plasma, and elevated plasma HDL-C level for healthy participants, and the pooled MD (95% confidence interval, CI) was −0.58 mmol/L (−0.86, −0.30 mmol/L), −0.22 mmol/L (−0.31, −0.13 mmol/L), −0.35 mmol/L (−0.54, −0.17 mmol/L), 0.13 mmol/L (0.06, 0.21 mmol/L), respectively. Baduanjin exercise also obviously decreased the levels of TG, LDL-C in plasma comparing with no treatment for patients, and the pooled MD (95% CI) was −0.30 mmol/L (−0.40, −0.19 mmol/L), −0.38 mmol/L (−0.63, −0.13 mmol/L), but there was not obvious to decrease plasma TC level or elevate plasma HDL-C level in patients with the pooled MD (95%CI), −0.39 mmol/L (−1.09, 0.31 mmol/L) and 0.22 mmol/L (−0.11, 0.55 mmol/L), respectively. In addition, the obvious advantage was not observed to modulate the blood lipid metabolism in comparing Baduanjin exercise with other exercises, regardless for health participants or patients. *Conclusion*. Studies indicated that Baduanjin exercise could significantly decrease the levels of TC, TG, LDL-C levels in plasma and elevate plasma HDL-C level for the healthy people. It also was helpful that Baduanjin exercise modulated the blood lipid metabolism for patients. Moreover, the Baduanjin exercise did not have an obvious advantage on modulating the lipid metabolism comparing with other exercises. But the evidence was uncertain because of the small sample size and low-methodological quality.

## 1. Introduction 

Hyperlipidemia is the condition of abnormally elevated levels of lipids (usually triglycerides and total cholesterol) and/or lipoproteins (usually low-density lipoprotein) in the blood, and is considered to be the most common reason of atherosclerosis [1, 2]. The results of a case control study conducted in 52 countries showed that abnormal lipid levels accounted for approximately 50% of the attributable risk for myocardial infarction (MI) in the population [3]. A prospective cohort study from different areas in China also indicated that 11.4% of the coronary deaths attributed to hypercholesterolemia [4]. Though hyperlipidemia is common in the general population, it also is regarded as a modifiable risk factor for cardiovascular disease because of its influence on atherosclerosis. The study found that the effective management of hyperlipidemia could obviously reduce cardiovascular morbidity and mortality substantially [5]. Predictive modeling in one study also suggested that every 10% increase in the prevalence of treatment of elevated low-density lipoprotein cholesterol (LDL-C) would lead to 8000 deaths prevented per year in those people aged <80 years [6]. For treatment of hyperlipidemia, statins and fibrates are highly effective, but may cause a markedly increased risk of myopathy and rhabdomyolysis [7].

In recent years, aerobic exercises, such as Chinese traditional exercise Baduanjin and Tai Ji, jogging, and yoga, are reported to be generally beneficial to healthy adults or the hyperlipidemia patients. Several studies found that they could significantly increase plasma high-density lipoprotein cholesterol (HDL-C) concentrations, or decrease plasma triglyceride (TC), triglycerides (TG), and LDL-C concentrations of the health people or patients by the regular exercise [8–11]. 

Baduanjin exercise is one of the most common forms of Chinese traditional exercises. It as a whole is broken down into eight separate exercises, which translated as eight pieces of brocade, and each one focusing on a different physical area and Qi meridian [12]. As a low-intensity aerobic exercise, the Baduanjin exercise not only was easy to learn but also have a less physical and cognitive demanding [10]. Therefore it has been taken as a popular and safe community exercise to promote health in China [13]. The modern basic researches showed that the Baduanjin exercise could improve the ability of lipoprotein lipase hydrolyzing triglycerides, promote synthesis of HDL-C by adding molecular weight of LDL receptor (LDL-R), and increase gene transcription and protein expression of LDL-R on the liver [14]. Furthermore, Baduanjin exercise could also enhance insulin sensitivity, and thus improved the blood lipid metabolism [15]. In addition, a low-intensity and long-time training of Baduanjin exercise could promote the consumption of lipid by elevating the ratio of the energy supply from lipid oxidation [16].

In the studies, although many of them thought that Baduanjin exercise was beneficial to modulate the blood lipid metabolism for the healthy participants or patients [1, 17], there was inconsistent opinion [18]. Moreover, these studies included so small sample size that we were unable to reach a positive conclusion. So the value of Baduanjin exercise for the blood lipid metabolism has not yet been proved. Consequently, the objective of this paper is to assess the effectiveness and adverse effects of Baduanjin exercise to favorably modulate the blood lipid concentration to healthy people and patients by undertaking a system review of trials, and address the apparent gap in the literature.

## 2. Methods

### 2.1. Data Sources

We searched the following databases from their inception to December 31, 2011 besides the Cochrane library (December, 2011): MEDLINE, EMBASE, Chinese Medical Literature Database (CBM), China National Knowledge Infrastructure (CNKI), Chinese Scientific Journal Database (VIP), China's Important Conference Papers Database, and China's Dissertation Database. The following search terms were used: “Baduanjin” individually in English databases, combined with “TC”, “TG”, “HDL”, “LDL”, “cholesterol”, “lipoproteins”, “lipid metabolism”, “high-density lipoprotein”, “low-density lipoprotein”, “triglycerides”, “hyperlipidemia”, and “hypercholesterolemia”, “hyperlipoproteinemia” in Chinese databases. In addition, the reference lists of all included papers were searched. Furthermore, the major domestic published journals, including Chinese Journal of Rehabilitation Medicine, Chinese Journal of Clinical Rehabilitation, and Chinese Journal of Sports Medicine, were also manually searched. 

Two reviewers (Mei LJ, Chen QY) conducted the literature searching independently. Then all articles searched were briefly selected after screening the title and abstract. And the eligibility articles were obtained and read in full text.

### 2.2. Study Selection and Data Extraction

All prospective controlled trials about Baduanjin compared with no treatment, routine treatment, or other exercises were included. Trials were excluded if the articles in included articles were duplicated publication and the data could not be extracted. The extracted primary outcome measures included TC, TG, HDL-C, and LDL-C. The secondary outcomes were the quality of life score (QoL score), body mass index (BMI), waist to hip ratio (WHR), body weight, and adverse events. Data were also extracted independently by the two reviewers, using a specifically designed data extraction form. 

### 2.3. Methodological Quality Assessment

The Cochrane collaboration's tool for the risk of bias were used to assess the quality of included articles, which based on six items: sequence generation, allocation concealment, blinding, incomplete outcome data, selective outcome reporting, and other sources of bias [19]. Each item was categorized as “low risk” of bias, “high risk” of bias, or “unclear risk” of bias according to the risk of material bias in the assessed trial. The quality of the eligibility trials was judged into low risk of bias (high quality) if all key items were low risk of bias, or unclear risk of bias if one or more key items was unclear risk of bias, or else high risk of bias (low quality). The two reviewers evaluated the quality of included trials, respectively. Disagreements were resolved by discussions. 

### 2.4. Data Analysis

All data were analyzed with the RevMan 5.1 software (Oxford, England) of The Cochrane Collaboration, and all *P* value were two-sided. If, on study design, participants, interventions, control, and outcome measures, the trials had a good homogeneity which evaluated by examining *I* ^2^, the results were pooled by using a fixed effect model. Otherwise, a random effect model was applied if the data could be used. The results were synthesized by using the relative risk (RR) with 95% confidence intervals (CI) for dichotomous data, while the mean differences (MD) with 95%CI were calculated for the continuous data. The funnel plot was used to explore the publication bias if sufficient studies were found.

## 3. Result

### 3.1. Study Description

**Figure 1:**
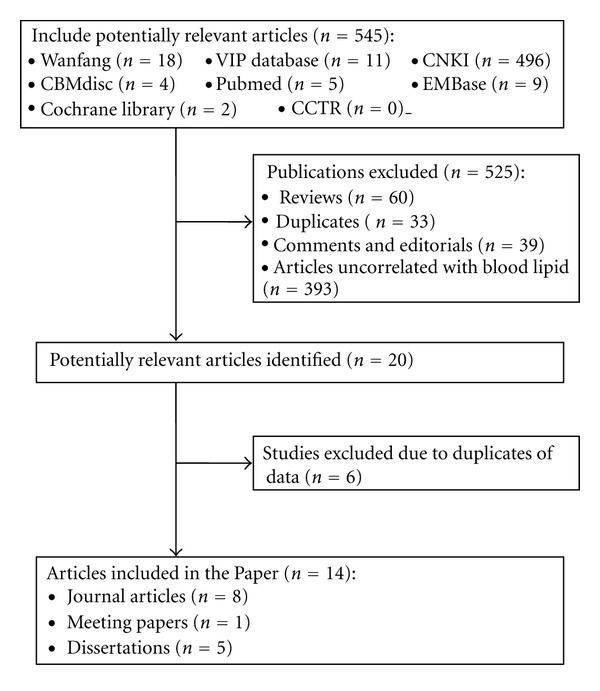
Flowchart of trial selection process.

A total of 545 literatures potentially relevant to Baduanjin exercise were searched after primary searches from eight databases. However, the majority of them were excluded after screening the titles and abstracts. About 20 full-text articles were finally assessed for eligibility. Of which 6 studies had to be excluded due to the duplicate publication. Therefore, the remaining 14 studies from 8 journal articles, 1 meeting paper, and 5 dissertations were included. A flow chart (Figure 1) illustrates the trial selection process.

The characteristics of included studies (14 studies with 20 comparisons) were listed in Supplement Table 1 of the Supplementary Material available online at doi:10.1155/2012/282131. A total of 859 participants including 48 hypertension, 45 schizophrenia, 331 type 2 diabetes, 50 hyperlipidemia, 23 overweight and 362 healthy participants were involved with age from 40 to 70 year. All included studies were conducted in China with published date from 2005 to 2011 year. Among these included studies, 10 studies were designed with RCTs, 1 study used before-after study design, and 3 studies used three- or four-arm study design (2 or 3 intervention groups compared with 1 control group). The types of intervention were classified as Baduanjin exercise alone or combined with other treatments (Baduanjin plus routine treatment or Baduanjin plus other exercises such as Liu Zi Jue, Wu Qin Xi, Walking, Jogging and so on). The intensity of Baduanjin exercise was unequal with 30 to 60 minutes per time, 1 to 2 times a week for 12 to 72 weeks. The control groups conducted routine treatment, or no treatment, or other exercises. The information about the sample size, participants, duration of intervention, and outcome assessment was presented in Supplement Table 1.

### 3.2. Methodological Quality

**Figure 2:**
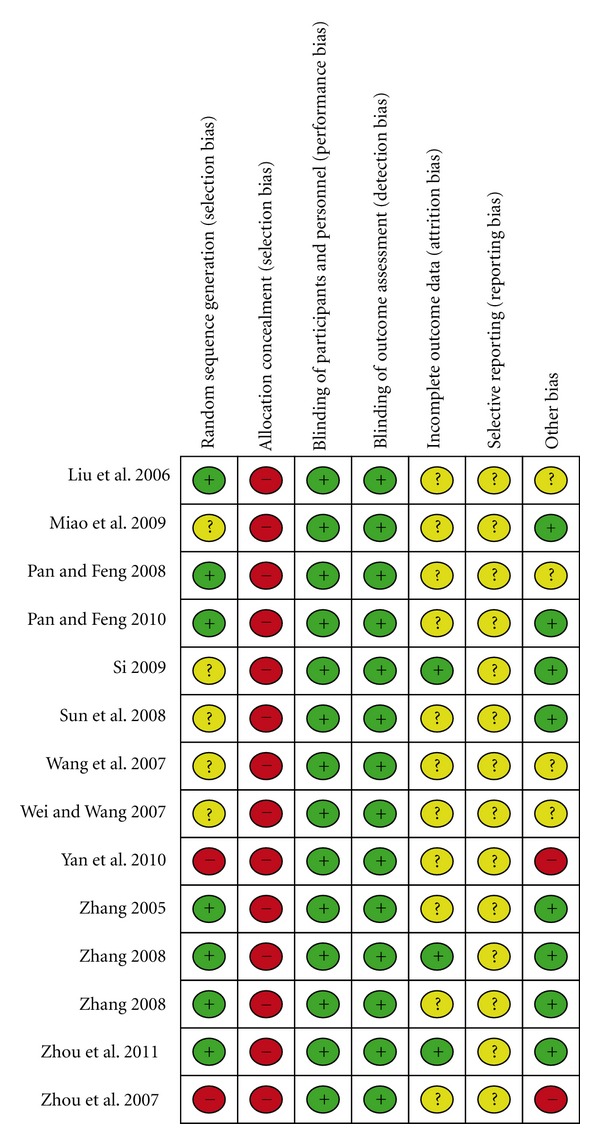
Risk of bias summary: review authors' judgements about each risk of bias item for each included study.

Only 5 studies stated the method of the sequence generation with random number table or computer software [8, 18, 20–22], while one study used patient preference [23]. None of them described allocation concealment. The blinding of participants and personnel was not possible because of the intervention types, and we judged this as having a low risk of bias because the outcomes were not likely to be influenced by lack of blinding. Blinding of outcome assessment was assessed to low risk of bias in all included studies because all outcome measurements were conducted by the outer lab assistants. Three studies reported the numbers of drop-out or withdraw, but all of them did not explain their reasons except for Si LY 2009 [24]. Moreover, none of the included studies used intention-to-treat analysis. There also were unclear on selective reporting outcome in the studies because of the inaccessibility to the study protocol. In a word, all included studies were assessed to be a poor methodological quality. The summary of bias risk for each study showed in Figure 2.

### 3.3. Effect of Baduanjin

Although some of trials illustrated no significant improvement effect, the consolidation data could claim certain improvement for blood lipid metabolism. The effect estimates were displayed in the Tables 1, 2, 3, and 4.

#### 3.3.1. TC

20 comparisons reported the effect of Baduanjin exercise alone or in combination with routine treatment for TC. Comparing with no treatment, Baduanjin exercise significantly reduced plasma TC levels for the healthy participants, and the pooled MD (95%CI) was −0.58 mmol/L (−0.86, −0.30 mmol/L). However, there was not statistical significant for patients, and the pooled MD (95%CI) was −0.39 mmol/L (−1.09, 0.31 mmol/L). Compared with other aerobic exercise group, participants in the Baduanjin group had no significant changes on the TC profile, the pooled MD with 95%CI was −0.07 mmol/L (−0.33, 0.19 mmol/L). Furthermore, comparing with the routine treatment or other exercises plus routine treatment, the significant decrease of plasma TC levels did not be found on Baduanjin plus routine treatment for the patients. The specific results were listed in Table 1.

#### 3.3.2. HDL-C

All the included trials were reported the changes of HDL-C after training Baduanjin exercise. Among them, there were a statistical significant increase of the plasma HDL-C levels for the healthy participants in comparing Baduanjin exercise alone with no treatment, and the pooled MD (95%CI) was 0.13 mmol/L (0.06, 0.21 mmol/L) with the *I* ^2^ value being 56%. Baduanjin exercise did not take more effective on elevating plasma HDL-C levels for the patients than other exercises, and the pooled MD (95%CI) was 0.22 mmol/L (−0.11, 0.55 mmol/L). 

Five studies compared Baduanjin exercise plus routine treatment with the same routine treatment for the patients. Two studies compared Baduanjin exercise with other exercises for the healthy participants. Three studies compared Baduanjin exercise plus routine treatment with other exercises plus the same routine treatment. All of these studies did not conclude that the intervention with Baduanjin exercise was better than the control intervention on elevating plasma HDL-C levels, and all *P* values were over 0.05. Those results were listed in Table 2. 

#### 3.3.3. TG

15 comparisons reported the changes of plasma TG levels. The treatment with Baduanjin exercise was found to significantly reduce the levels of TG comparing with no treatment for the healthy participants or the patients, and the pooled MD (95%CI) were −0.22 mmol/L (−0.31, −0.13 mmol/L), −0.30 mmol/L (−0.40, −0.19 mmol/L), respectively. The patients with Baduanjin exercise plus routine treatment did not have significantly lower plasma TG levels than ones treated with the same routine treatment. Likely, a statistically significant difference of plasma TG level was not revealed between the patients treated with Baduanjin exercise plus routine treatment and with other exercise plus the same routine treatment. For the healthy participants, the Baduanjin exercise did not have a more obvious ability to reduce the plasma TG levels than the other exercises, as showed in Table 3. 

#### 3.3.4. LDL-C

As shown in Table 4, nineteen comparisons illustrated the LDL-C profile between the Baduanjin group and other control groups. For the healthy participants, Nine trials compared Baduanjin exercise with no treatment, the statistically significant decrease on plasma LDL-C levels could be found, and the pooled MD (95%CI) was −0.35 mmol/L (−0.54, −0.17 mmol/L) with the moderate heterogeneity (*I* ^2^ = 47%, *P* = 0.05). Two trials compared Baduanjin exercise with other exercises, the statistically significant difference was not observed. For the patients, Baduanjin exercise could reduce the plasma LDL-C levels comparing with no treatment, and the pooled MD (95%CI) was −0.38 mmol/L (−0.63, −0.13 mmol/L). But comparing with the routine treatment or other exercises plus the routine treatment, Baduanjin exercise plus the routine treatment did not have an obvious advantage on reducing plasma LDL-C levels, while the statistical significance was not observed. 

#### 3.3.5. The Safety

None of the included studies reported the safety of Baduanjin exercise.

#### 3.3.6. The Secondary Outcomes

The secondary outcome measures were reported in five selected studies with 7 comparisons. QoL score including physiology score, psychological score, social score, and treatment score were reported in 2 studies with 3 comparisons. BMI and WHR were measured in 4 studies, and 2 studies respectively. In addition, 3 studies reported the body weight of participants.

Comparing with the routine treatment, Baduanjin exercise plus the same routine treatment could improve the physiology score of patients, and the pooled MD (95%CI) was −9.53 (−16.5, −2.2), the other scores including psychological score, social score, and treatment score had no obvious changes. Baduanjin exercise could reduce the BMI and WHR of healthy adults comparing with no other exercises, and the pooled MD (95%CI) were −2.46 (−3.69, −1.23), and −0.05 (−0.09, −0.02). The body weight had significant decrease for patients in comparing Baduanjin exercise plus routine treatment with other exercise plus the same routine treatment, and their pooled MD (95%CI) was −4.5 kg  (−7.65, − 1.35 kg). The remaining variables were not found the significant changes.

### 3.4. Sensitivity Heterogeneity

In the study, several comparisons showed obvious heterogeneity among the analyzed studies. In order to find out the source of heterogeneity, we conducted the sensitivity analysis for them which their *I* ^2^ values exceeded 50% or *P*_heterogeneity_ < 0.05. The results were listed in Table 5. In the comparisons of Baduanjin exercise plus routine treatment versus the same routine treatment for plasma TC levels or HDL-C levels of the patients, the pooled results were inversed from a nonstatistical significance to an obvious statistical significance, and the *I* ^2^ values were decreased obviously after the dubious studies were removed. Moreover, the pooled MD was also changed to an statistical significance in the comparison of Baduanjin exercise plus routine treatment with other exercise plus the same routine treatment for LDL-C levels of the patients with the *I* ^2^ values decreasing to 53%. The substantive changes were not found in the remaining sensitivity analysis.

### 3.5. Publication Bias

We were unable to explore the publication bias with the funnel plot because every comparison was less than 10 studies.

## 4. Discussion

In this systematic review, 14 studies with 21 comparisons accounting for 362 healthy participants and 497 patients were identified. 5 studies were found to have the definitive randomization, but none of them described the allocation concealment. The bias of selective reporting outcome was uncertain because of the inaccessibility to the study protocol. Therefore, as a whole, the included studies were of low-methodological quality.

In the primary outcomes, the results from 3 studies with 8 comparisons involving 224 healthy participants for TC levels [1, 10, 25], 2 studies [1, 25] with 4 comparisons including 136 healthy participants for TG levels and 4 studies [1, 10, 24, 25] with 9 comparisons involving 284 healthy participants for LDL-C levels showed that Baduanjin exercise could obviously decrease the plasma TC, TG, LDL-C levels of the healthy adults comparing with controls without any other exercises. Furthermore, the result from 4 studies with 9 comparisons involving 284 healthy participants also revealed that Baduanjin exercise could elevate the plasma HDL-C levels for healthy participants comparing with no-exercise controls. It indicated that Baduanjin exercise was beneficial to modulate the lipid metabolism for healthy adults. Likely, comparing with no treatment, Baduanjin exercise also obviously decreases the plasma TG, LDL-C levels for the patients. But the evidence was uncertain because of the small sample size and substantial heterogeneity. Moreover, comparing with other exercises, Baduanjin exercise did not have an obvious advantage on modulating the lipid metabolism regardless for healthy adults or patients.

The secondary outcomes including in QoL, body weight, WHR, and BMI were merely reported in five selected studies. QoL score in the Baduanjin group were shown more significant improvement than in the control group in two consolidated trials [18, 20]. One study [22] had a significant decrease in body weight. Another study also found [21] that Baduanjin exercise can significantly decrease the WHR and BMI of participants. 

Although the result of meta-analysis showed a statistically significant improvement to the blood lipid metabolism of the participants, the definite conclusion cannot be drawn from this paper because of the following limitations. Firstly, the small sample size of studies ranged from 12 to 126, and none of them reported sample size calculations as suggested by the CONSORT statement. Secondly, a few studies reported the method of randomization, allocation concealment, the drop-out, and withdraw, which resulted in the low-methodological quality. Thirdly, the variable mean age, different diseases of the patients, different exercise programs in the control group, and discrepancy of pre-exercise blood lipid levels could lead to the substantial heterogeneity which could influence the pooled outcomes. Furthermore, we were unable to explore the publication bias using the funnel plot because each comparison had insufficient studies (less than 10), but the publication bias might be existed in this paper despite the positive and non-positive studies had already been published. In addition, language bias could be created because all the included studies are Chinese.

There were no reviews on the effects of Baduanjin exercise improving the blood lipid disorders. However, about the effects of exercises, especially aerobic exercises such as jogging, walking, and tai chi, many researches have been done recently [26, 27]. Although Jorge F. held that the quantitative effect of physical activity and exercises training on lipid and lipoprotein profiles is often small for patients with (or at risk for) cardiovascular disease, stroke, and other types of atherosclerotic vascular disease [26]. A meta-analysis on blood lipids and lipoproteins of 66 controlled trials with 2925 participants illustrated a large decrease in TC, TG, LDL-C, and TC/HDL after physical exercise training [27]. In the current paper, studies tested that Baduanjin exercise for healthy adults were also observed to decrease plasma TC, TG, LDL-C levels, and increase plasma HDL-C levels comparing with the blank control. Furthermore, it is helpful to the patients after training Baduanjin exercise in comparison of no treatment. However, comparing with other exercise, the obvious advantage was not observed to improve the blood lipid metabolism of health participants or patients. 

## 5. Conclusions 

In conclusion, we can hold that Baduanjin exercise comparing with no treatment could modulate the blood lipid profile by decreasing plasma TC, TG, and LDL-C levels and increasing plasma HDL-C levels of the participants. Comparing with other exercises, this obvious advantage was not found on the Baduanjin exercise modulating the lipid metabolism. However, the conclusions were uncertain because of the low-methodically quality in the included studies. So it currently needs to be one ongoing large sample size and high-quality prospective controlled trials, which can be testifying the effectiveness of Baduanjin exercise. As far as the definite benefits of Baduanjin exercise have been confirmed, it can be applied broadly throughout the community.

## Supplementary Material

Excluded studies with reasons, search strategy and Baduanjin exercise's brief steps were appended in the supplementary materials.
Supplementary materials: Supplement Table 1: Characteristics of included publications, Supplement Table 2: Excluded studies with reasons, Appendices A. Search strategy, Appendices B. Baduanjin exercise's brief steps

## Figures and Tables

**Table 1:** The pooled results of Baduanjin exercise for plasma TC levels in healthy participants or clinical patients.

Participants	Number of comparisons	*n*/T	*n*/C	MD (95% CI) (mmol/L)	*P* value	*I* ^2^ (%)	*P*_ heterogeneity_
Baduanjin + routine versus routine treatment							
Clinical patients	5	136	136	−0.56 (−0.74,0.37)	*P* = 0.15	86	*P* < 0.00001
Baduanjin versus no treatment							
Clinical patients	2	38	35	−0.39 (−1.09, 0.31)	*P* = 0.28	77	*P* = 0.04
Healthy participants	8	110	114	−0.58 (−0.86, −0.3)	*P* < 0.0001	41	*P* = 0.1
Baduanjin versus other exercises							
Healthy participants	2	49	47	−0.07 (−0.33,0.19)	*P* = 0.6	0	*P* = 0.6
Baduanjin + routine versus other exercises + routine treatment							
Clinical patients	3	114	106	−0.01 (−0.25,0.24)	*P* = 0.96	68	*P* = 0.04

T: The treatment group, C: The control group.

**Table 2:** The pooled results of Baduanjin exercise for plasma HDL-C level in healthy participants or clinical patients.

Participants	Number of comparisons	*n*/T	*n*/C	MD (95% CI) (mmol/L)	*P* value	*I* ^2^ (%)	*P*_ heterogeneity_
Baduanjin + routine treatment versus routine treatment							
Clinical patients	5	136	136	0.04 (−0.1, 0.18)	*P* = 0.59	73	*P* = 0.006
Baduanjin versus no treatment							
Clinical patients	2	38	35	0.22 (−0.11, 0.55)	*P* = 0.19	92	*P* = 0.0005
Healthy participants	9	140	144	0.13 (0.06, 0.21)	*P* = 0.0006	56	*P* = 0.02
Baduanjin versus other exercises							
Healthy participants	2	49	47	−0.09 (−0.25, 0.06)	*P* = 0.25	19	*P* = 0.27
Baduanjin + routine versus other exercises + routine treatment							
Clinical patients	3	114	106	0.00 (−0.06, 0.07)	*P* = 0.96	0	*P* = 0.62

T: The treatment group, C: The control group.

**Table 3:** The pooled results of Baduanjin exercise for plasma TG level in healthy participants or clinical patients.

Participants	Numbers of comparisons	*n*/T	*n*/C	MD (95% CI) (mmol/L)	*P* value	*I* ^2^ (%)	*P*_ heterogeneity_
Baduanjin + routine treatment versus routine treatment							
Clinical patients	4	112	112	−0.77 (−1.97, 0.42)	*P* = 0.2	94	*P* < 0.00001
Baduanjin versus no treatment							
Clinical patients	2	38	35	−0.30 (−0.40, −0.19)	*P* < 0.00001	42	*P* = 0.19
Healthy participants	4	66	70	−0.22 (−0.31, −0.13)	*P* < 0.00001	0	*P* = 0.88
Baduanjin versus other exercises							
Healthy participants	2	49	47	−0.21 (−0.51, 0.1)	*P* = 0.18	90	*P* = 0.001
Baduanjin + routine treatment versus other exercises + routine treatment							
Clinical patients	3	114	106	0.1 (−0.14, 0.35)	*P* = 0.41	0	*P* = 0.9

T: The treatment group, C: The control group.

**Table 4:** The pooled results of Baduanjin exercise for plasma LDL-C level in healthy participants or clinical patients.

Group	Numbers of comparisons	*n*/T	*n*/C	MD (95% CI) (mmol/L)	*P* value	*I* ^2^ (%)	*P*_ heterogeneity_
Baduanjin + routine treatment versus routine treatment							
Clinical patients	3	88	88	0.14 (−0.03, 0.31)	*P* = 0.1	60	*P* = 0.08
Baduanjin versus no treatment							
Clinical patients	2	38	35	−0.38 (−0.63, −0.13)	*P* = 0.002	0	*P* = 0.62
Healthy participants	9	140	144	−0.35 (−0.54, −0.17)	*P* = 0.0002	47	*P* = 0.05
Baduanjin versus other exercises							
Healthy participants	2	49	47	0.08 (−0.07, 0.23)	*P* = 0.28	74	*P* = 0.05
Baduanjin + routine treatment versus other exercises + routine treatment							
Clinical patients	3	114	106	0.08 (−0.43, 0.58)	*P* = 0.76	88	*P* = 0.0003

T: The treatment group, C: The control group.

**Table 5:** Sensitivity analysis of the effect of Baduanjin exercise to clinical patients or healthy participants.

Before sensitivity analysis					After sensitivity analysis			
Comparisons	MD (model) 95% CI	*P* value	*I* ^2^ (%)	Method of sensitivity analysis	Comparisons	MD (model) 95% CI	*P* value	*I* ^2^ (%)
Baduanjin + routine treatment versus routine treatment for TC in clinical patients								
5	−0.56 (random) (−0.74, 0.37)	*P* = 0.15	86	Remove Zhang 2005 and Zhang 2008	3	−0.83 (fixed) (−1.04, −0.62)	*P* < 0.00001	0
Baduanjin + routine treatment versus other exercises for TC in clinical patients								
3	−0.01 (random) (−0.25, 0.24)	*P* = 0.96	68	Remove Zhang 2008	2	−0.17 (fixed) (−0.45, 0.11)	*P* = 0.23	0
Baduanjin + routine treatment versus routine treatment for HDL-C in clinical patients								
5	0.04 (random) (−0.1, 0.18)	*P* = 0.59	73	Remove Zhang 2008	4	0.12 (fixed) (0.06, 0.18)	*P* < 0.0001	51
Baduanjin versus no treatment for HDL-C in health adults								
9	0.13 (random) (0.06, 0.21)	*P* = 0.0006	56	Remove Liu et al. 2006	8	0.16 (fixed) (0.11, 0.21)	*P* < 0.00001	19
Baduanjin + routine treatment versus routine treatment for TG in clinical patients								
4	−0.77 (random) (−1.97, 0.42)	*P* = 0.2	94	Remove Wang et al. 2007	3	−0.16 (fixed) (−0.49, 0.17)	*P* = 0.34	0
Baduanjin + routine treatment versus other exercises plus routine treatment for LDL-C in clinical patients								
3	0.08 (random) (−0.43, 0.58)	*P* = 0.76	88	Remove Zhou et al. 2011	2	0.29 (fixed) (0.05, 0.53)	*P* = 0.02	53
